# Quantitative Assessment of *Mycoplasma* Hemadsorption Activity by Flow Cytometry

**DOI:** 10.1371/journal.pone.0087500

**Published:** 2014-01-30

**Authors:** Luis García-Morales, Luis González-González, Manuela Costa, Enrique Querol, Jaume Piñol

**Affiliations:** 1 Institut de Biotecnologia i Biomedicina and Departament de Bioquímica i Biologia Molecular, Universitat Autònoma de Barcelona, Bellaterra, Barcelona, Spain; 2 Servei de Cultius Cel·lulars, Producció d’Anticossos i Citometria, Universitat Autònoma de Barcelona, Bellaterra, Barcelona, Spain; University of Ottawa, Canada

## Abstract

A number of adherent mycoplasmas have developed highly complex polar structures that are involved in diverse aspects of the biology of these microorganisms and play a key role as virulence factors by promoting adhesion to host cells in the first stages of infection. Attachment activity of mycoplasma cells has been traditionally investigated by determining their hemadsorption ability to red blood cells and it is a distinctive trait widely examined when characterizing the different mycoplasma species. Despite the fact that protocols to qualitatively determine the hemadsorption or hemagglutination of mycoplasmas are straightforward, current methods when investigating hemadsorption at the quantitative level are expensive and poorly reproducible. By using flow cytometry, we have developed a procedure to quantify rapidly and accurately the hemadsorption activity of mycoplasmas in the presence of SYBR Green I, a vital fluorochrome that stains nucleic acids, allowing to resolve erythrocyte and mycoplasma cells by their different size and fluorescence. This method is very reproducible and permits the kinetic analysis of the obtained data and a precise hemadsorption quantification based on standard binding parameters such as the dissociation constant *K*_d_. The procedure we developed could be easily implemented in a standardized assay to test the hemadsorption activity of the growing number of clinical isolates and mutant strains of different mycoplasma species, providing valuable data about the virulence of these microorganisms.

## Introduction

Mycoplasmas are a wide group of microorganisms characterized by the absence of cell wall and are closely related to Gram-positive bacteria. These microorganisms possessing small streamlined genomes and a low number of metabolic pathways [1] probably evolved as a consequence of their parasitic lifestyle. Many mycoplasmas are pathogens of humans and a wide range of animals, and most of them adhere to host cells in the first stages of infection [2], [3], being this step essential for further colonization [4]. Some of these adherent mycoplasmas have developed highly complex polar structures assisting cells in the attachment to target tissues [5], [6]. These specialized structures also propel the cells when they glide across solid surfaces [7], [8] and have a pivotal role on virulence [9], [10].

*Mycoplasma genitalium* is a human pathogen that is the causative agent of non-chlamidial and non-gonococcal urethritis [11]. Besides constituting an appealing model for minimal cell and synthetic biology studies, this microorganism is also an excellent subject to investigate the adhesion mechanisms both at molecular [6] and clinical level [12], [13], [14]. A collection of *M. genitalium* mutants showing attachment deficiencies is now available [6], [15] and the accurate measurement of hemadsorption (HA) activity is essential to obtain data about the function of the different proteins involved in cell adhesion. In addition, measuring the HA of the growing number of clinical isolates [16], [17] may provide important clues about the infective mechanisms of this microorganism. A standardized assay to quantify HA would also benefit the growing number of studies in many other related mycoplasmas with special relevance of those involved either in human or animal health. Furthermore, the use of a standardized assay in clinical practice would help in the prognosis of mycoplasma infections, which is especially relevant in infections by outbreak strains occurring worldwide [18], [19], [20].

Different methods are currently available to quantify the HA of mycoplasmas [15], [21], [22]. However, they are very expensive, time consuming or not reproducible enough to be used in standardized clinical assays. Given these limitations, measuring HA by an approach based in flow cytometry (FC) could be interesting in terms of accuracy, reproducibility and speed. However, there are relatively few works dealing with FC and mycoplasmas, probably because of the small size of mycoplasma cells that may discourage, at first glance, their analysis by FC. Despite this, there is an increasing number of works showing that FC is an accurate method to detect and quantify mycoplasma cells in broth medium [23], [24] or in natural samples [25]. FC has been used also to quantify the effect of several antibacterial agents [26], [27] and to detect hemotropic mycoplasmas on red blood cells (RBCs) samples [28]. Here we show that FC could be used to measure rapidly and accurately the HA activity of mycoplasmas in the presence of SYBR Green I, a vital fluorochrome that stains nucleic acids, allowing to resolve RBCs and mycoplasma cells by their different size and fluorescence. This method also permits the kinetic analysis of the obtained data, allowing a precise HA quantification based on standard parameters such as the dissociation constant *K*_d_. An additional advantage of the kinetic analysis is the provision of a reproducible method to compare the HA activity of strains from different mycoplasma species.

## Materials and Methods

### Red Blood Cells, Mycoplasma Strains and Culture Conditions

*M. genitalium* wild type G37 and its isogenic mutant strains mg218^−^, mg317^−^ and mg191^−^ exhibiting attachment deficiencies [6], [15] and *Mycoplasma penetrans* GTU-54 were grown in 75 cm^2^ tissue culture flasks (TPP) containing 20 mL of 0.22 µm filtered SP4 medium [29] at 37°C under 5% CO_2_ until reaching the mid-log phase of growth as previously described [30]. *Mycoplasma hyopneumoniae* J strain was grown in suspension using 20 mL of Friis medium [31] in 50 mL culture tubes (TPP) until reaching the mid-log phase of growth. Both adherent mycoplasma cells scrapped off the tissue culture flasks and cells growing in suspension were recovered by centrifugation 20 minutes at 20 000 *g* and resuspended in 0.5 mL of culture medium. Mycoplasma cells were passed ten times through a syringe with a 25G needle before being titrated and submitted to the HA assay. RBCs from 0.250 mL samples of peripheral blood of a human healthy donor were washed three times with 10 mL of Dulbecco’s phosphate buffered saline containing 0.9 mM CaCl_2_ and 0.49 mM MgCl_2_ (PBSCM, Sigma-Aldrich Corp.) and resuspended in 25 mL of the same buffer. Special care was taken to remove the upper layer of white blood cells (WBCs) in the RBCs pellet. The stock of RBCs obtained by this method was stored on ice and used in the interval of four hours in the HA assay. Written informed consent was obtained from the blood donor. All the procedures were under the guidelines established by the Human and Animal Experimentation Ethics Committee (CEEAH) of UAB and approved by this committee.

### Cell Titration and Flow Cytometry Analysis

The different mycoplasma samples were first diluted 1∶200 in culture medium. Then, 20 µL of these suspensions were diluted again in 500 µL of PBSCM and stained with the fluorochrome SYBR Green I (Molecular Probes) at a 1∶10 000 (v/v) dilution from the commercial stock for 20 min protected from light. Similarly, the stock of RBCs was diluted 1∶150 in PBSCM and stained also with SYBR Green at a 1∶10 000 dilution. At the same time, a sample of 500 µL of PBSCM containing 20 µL of SP4 medium was stained by the same procedure. Samples were titrated by FC using a FACSCalibur (Becton Dickinson) equipped with an air-cooled 488 nm argon laser and a 633 nm red diode laser. Side-angle-scatter (SSC-H), green fluorescence (FL1-H detector, 530/30 filter) and red autofluorescence (FL3-H detector, 670LP filter) were used to quantify the different cell types used. FC data was acquired in four-decade logarithmic scale and the samples were measured during 90 seconds at the lowest flow rate (12 µL min^−1^) to increase the resolution of readings. Total fluorescence units of FL1-H were calculated by multiplying the FL1-H mean value by the total number of events in the analyzed region. The primary threshold was set at 11 units of SSC-H and the secondary threshold at 27 units of FL1-H. FC data were analyzed with the CellQuest-Pro software (Becton Dickinson) and contour plots were made using FACSDiva software (Becton Dickinson). To identify and titrate mycoplasma cells, several dilutions of the fresh mycoplasma stock containing the same amount of SP4 medium were used. The viability of mycoplasma cells was assessed in a sample of SYBR Green I stained mycoplamas by adding propidium iodide (PI) to the mixture at a final concentration of 2 µg mL^−1^. Non viable cells were expected to show increased FL3-H fluorescence values when analyzed by FC. To define the region containing non-viable mycoplasmas, a mycoplasma sample containing SYBR Green I and propidium iodide was permeabilized with 0.015% Triton X-100 (Fluka) just before submitting the sample to FC analysis.

### Hemadsorption Reaction

A fixed amount of mycoplasma cells (about 3·10^6^ FL1-H fluorescence units) was incubated with increasing amounts of RBCs (from 0 to 10^7^ FC events) in 1 mL of PBSCM. It was found essential for reproducible results that mycoplasma culture medium in the HA reaction was at least 5% (v/v). Samples were gently mixed end-over-end for 40 min at 37°C and then SYBR Green was added at a 1∶10 000 dilution and further incubated 20 min protected from light. The fraction of free mycoplasma cells remaining after the HA reaction was quantified by FC as described above and plotted versus the amount of RBCs. HA data for each strain and species was obtained at least from three independent biological replicates. Plots and data fitting to inverse Langmuir isotherm curves were performed by iteration using KaleidaGraph software (Synergy). After FC analyses, some of the samples of RBCs mixed with mycoplasmas were examined by phase contrast and epifluorescence using a Nikon Eclipse TE2000e microscope. Pictures obtained with a Digital Sight-SMC Nikon camera were processed using the NIS-Elements BR software. A detailed working protocol of the whole HA assay procedure is provided in supporting Protocol S1.

## Results

### Cell Titration and Biomass Estimation

**Figure 1:**
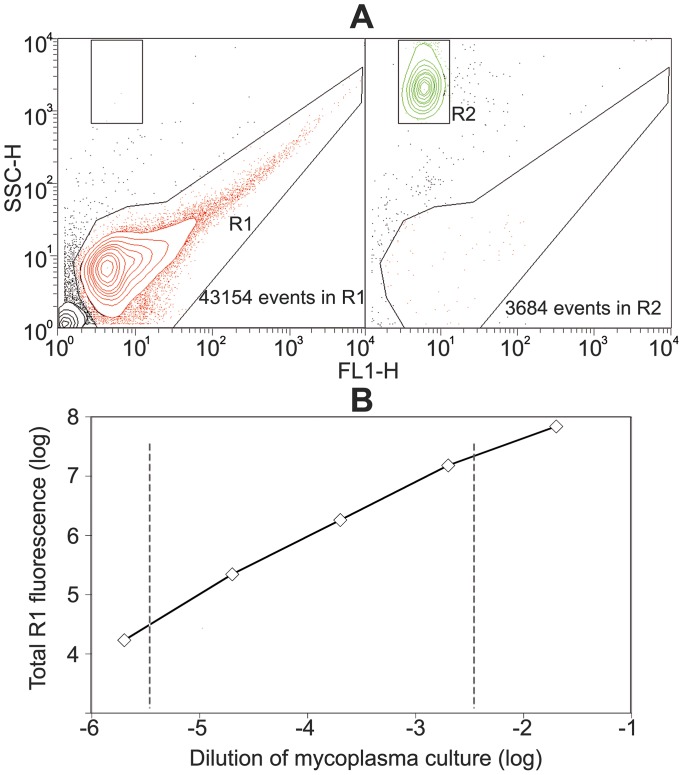
Titration of mycoplasma cells by flow cytometry. (A) Dual parameter dot plot of SSC-H (Side-angle-scatter) versus FL1 (SYBR Green fluorescence) showing events from a *M. genitalium* G37 population (R1 region containing 4·10^4^ events and 8·10^5^ arbitrary units of total fluorescence) and RBCs (R2 region containing 3.6·10^3^ events). (B) Total fluorescence of the events in R1 region from serial dilutions of a *M. genitalium* culture showing a lineal distribution over a three log dilution range, which is indicated by dashed lines. Total fluorescence units of FL1-H were calculated by multiplying the FL1-H mean value by the total number of events in the analyzed region.

Once stained with SYBR Green and analyzed by FC, events from mycoplasma cells were very heterogeneous and are enclosed in a wide zone (R1, Figure 1A) of FC events. Most of mycoplasma cells raised events of low SSC-H and SYBR Green fluorescence values but there were also a variable number of cell aggregates generating events with increased values for both parameters. On the other hand, and in agreement with their large size, RBCs appear in a discrete region of events (R2, Figure 1A) with high SSC-H values but a low SYBR Green fluorescence due to their low content of nucleic acids. To test that mycoplasma cells were efficiently stained with SYBR Green I, a sample of stained mycoplasmas was examined by phase contrast and epifluorescence microscopy showing that virtually all mycoplasma cells were stained with this dye (Figure S1).

RBCs stock was tittered by counting the events showing high SSC-H values but a low fluorescence when stained with SYBR Green [32]. However, tested mycoplasma cells were very prone to form cell aggregates and therefore the counting of the number of FC events is not a good method for assessing the biomass of these cells. We found that the total SYBR Green fluorescence of the mycoplasma population provides a better estimation of cell biomass since SYBR Green fluorescence is directly correlated with the amount of nucleic acids in the analyzed sample. Total SYBR Green fluorescence also shows a linear response spanning about three logs when examining different dilutions of *M. genitalium* cultures (Figure 1B). All the mycoplasma samples used in the HA assay had a total SYBR Green fluorescence values among the limits of the linear region in Figure 1B.

### FC Analysis of Mycoplasma HA

**Figure 2:**
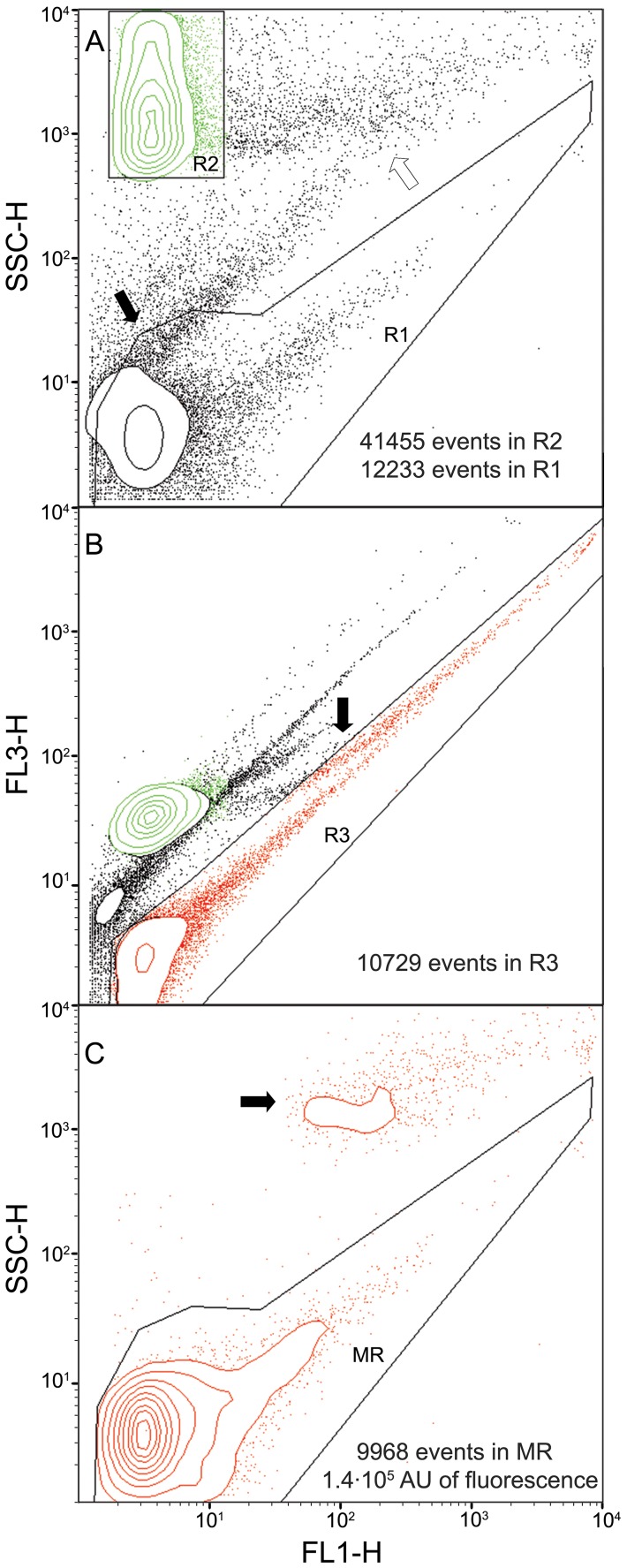
Resolution of cell populations after the HA reaction containing RBCs, RBCs with attached mycoplasmas and free mycoplasmas. (A) Overview of the cell mixture in a dual plot of SSC-H vs. FL1-H fluorescence. R2 region includes RBCs and R1 region contains the free mycoplasmas after the HA reaction. The solid arrow points to an area where the debris from the culture medium are overlapping with mycoplasmas in the R1 region and the empty arrow indicates an area with debris overlapping the R2 region. (B) Dual plot of FL3-H vs. FL1-H fluorescence allowing the separation of the free mycoplasmas from debris due to the intrinsically high autofluorescence of these particles. Note that R3 region includes the free mycoplasmas after the HA reaction but also contains a significant fraction of RBCs with attached mycoplasmas. The arrow points to these RBCs with attached mycoplasmas invading the R3 region. (C) A new dual parameter plot of SSC-H vs. FL1-H showing only the events in R3 region finally resolves the free mycoplasmas (MR region) from the RBCs with attached mycoplasmas, pointed with an arrow. Total fluorescence of free mycoplasmas in the MR region can now be precisely quantified and used in the subsequent calculation of the HA parameters *K*_d_ and *B*_max_.

After incubating RBCs and mycoplasma cells, it was noticeable a decrease in the total fluorescence of the mycoplasma region R1 and a shift to higher fluorescence values of the events in the RBC region R2 (Figure 2A) consistent with the attachment of mycoplasmas to RBCs. However, it was also evident a new population of events from particles generated while the FC samples were incubated with mild shaking. These particles were also apparent even in the absence of mycoplasmas and RBCs (Figure S2) suggesting that these particles were derived from the mycoplasma culture medium added to the HA reaction. The size and the total fluorescence of this new population was very variable among different experiments, with events overlapping R1 and R2 regions (Figure 2A) and precluding the accurate measurement of the fluorescence in these regions. Fortunately, events of this new population had an intrinsically high red autofluorescence (Figure 2B) providing a way to discriminate them from events of mycoplasma cells, which remained grouped in a new R3 region. Double gating of R1 and R3 regions allowed to construct new region MR containing only the events from mycoplasma cells (Figure 2C), endowing a simple method to quantify in a reproducible way the total fluorescence of free mycoplasmas remaining after the HA reaction. Furthermore, we investigated the presence of WBCs in the RBCs stock and if these nucleated cells may interfere with the mycoplasma quantification in the HA assay. We detected a very small amount of WBCs in the preparation of RBCs, with no events overlapping the MR region of mycoplasma cells (Figure S3) indicating that WBCs were not interfering the HA assay. In addition, some mycoplasma samples were also examined using a double staining by adding propidium iodide to quantify the frequency of dead cells. Only 4% of mycoplasma cells were stained with propidium iodide (Figure S4), indicating that most of cells remained viable at least for two hours in the settings used to perform the HA reaction.

**Figure 3:**
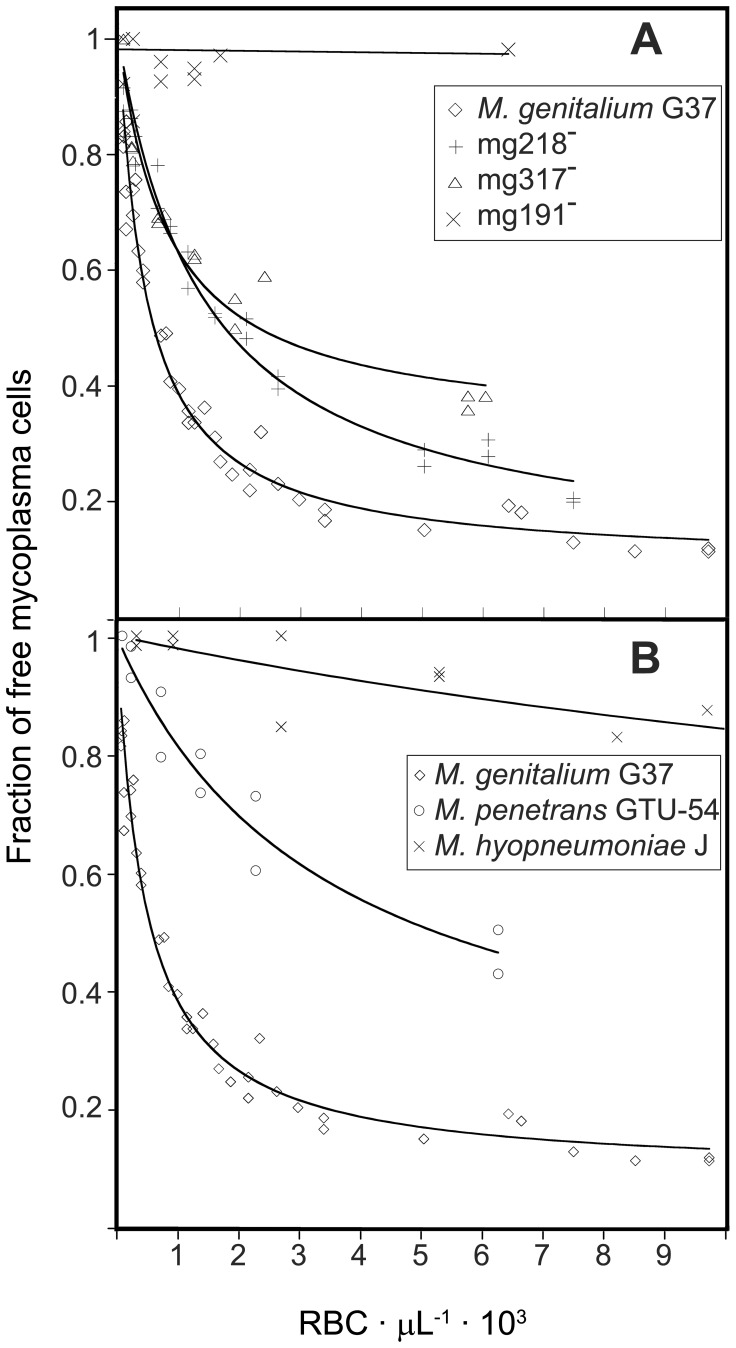
Inverse Langmuir plots of HA assays containing a fixed amount of mycoplasma cells and increasing amounts of RBCs. The fraction of free mycoplasma cells was calculated from the total FL1-H fluorescence values in the MR region (Figure 2) of each HA reaction taking as reference the total FL1-H fluorescence in a mycoplasma sample without RBCs. (A) Plots from HA assays containing *M. genitalium* G37 strain and its isogenic mutant strains mg218^−^, mg317^−^ and mg191^−^. (B) Plots from HA assays performed with different mycoplasma species.

**Figure 4:**
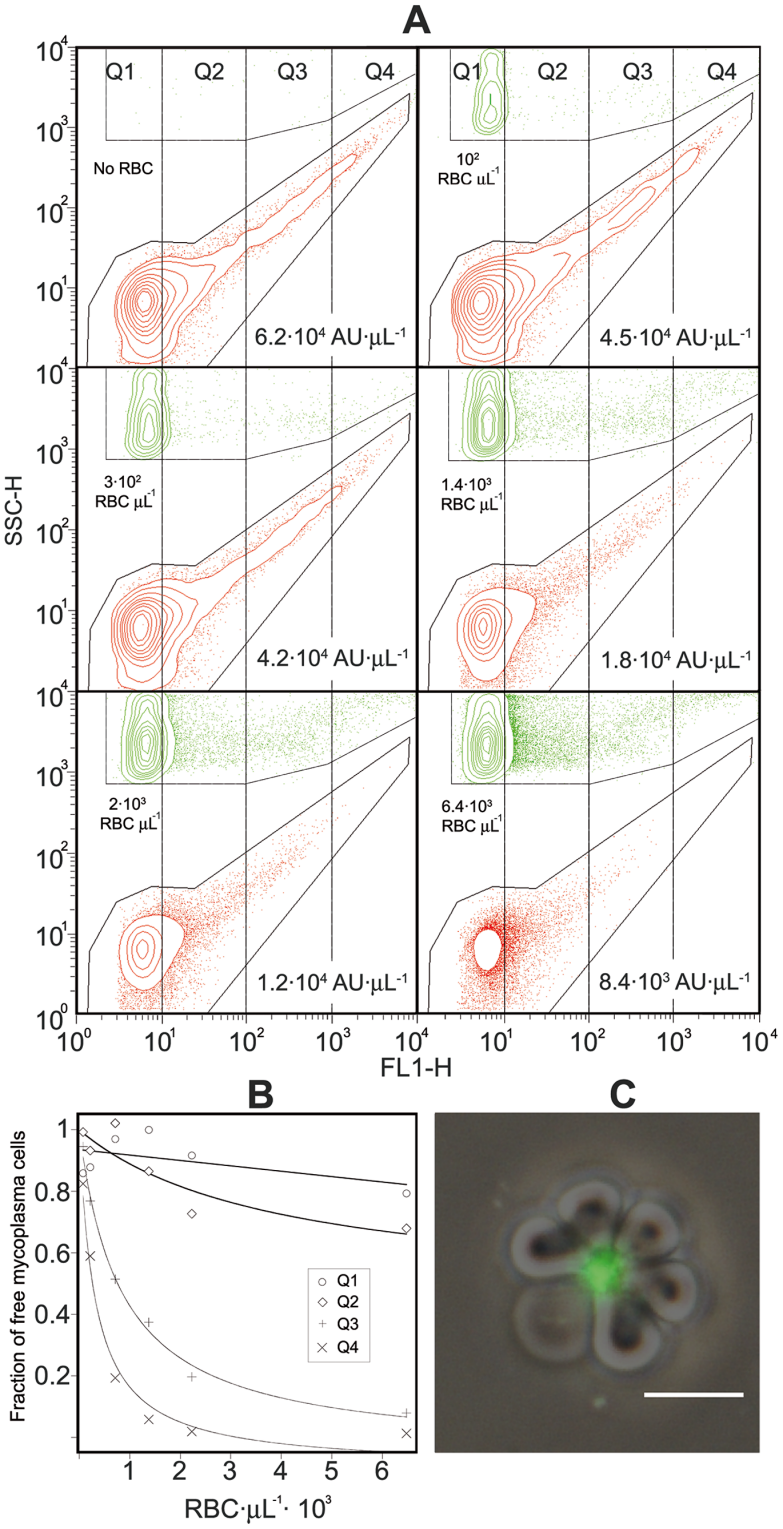
Differential binding properties of mycoplasma cell aggregates and singly mycoplasma cells. (A) Dual parameter dot plots of SSC-H vs. FL1-H fluorescence from several HA reactions showing the events in MR and R2 regions. MR region was defined as described in Figure 2. R2 region contains the events from free RBCs and RBCs containing attached mycoplasmas. These plots contain a fixed amount of *M. genitalium* cells and increasing amounts of RBCs. Each dot plot was divided into four separate quartiles (Q1 to Q4) and the total fluorescence in MR region of each quartile was used to construct the respective inverse Langmuir plots in panel B. (C) Representative micrograph showing RBCs and mycoplasmas after the assay. The phase contrast picture is merged with the SYBR Green I epifluorescence picture, which is false colored to green. Bar is 10 µm. Note that most of SYBR green stained mycoplasmas are in big particles much larger than single cells, usually measuring 0.5–0.8 µm.

The first experiments to quantify the HA reaction were conducted using a fixed amount of RBCs, increasing amounts of mycoplasma cells and measuring the shift to higher fluorescence values of the events in the RBC region. However, this approach has two severe drawbacks. By one hand, when using increasing amounts of mycoplasma cells, regions R1 and R2 were gradually closer and became eventually overlapped. On the other hand, events from the particulate material of the culture medium are very prone to overlap with R2 region as indicated above and this problem was exacerbated at the highest concentrations of mycoplasma cells (Figure S5). In consequence, we designed a new HA assay using a fixed amount of mycoplasma cells, increasing amounts of RBCs and measuring the free mycoplasmas remaining after the HA reaction. Using these conditions, the amount of free mycoplasmas decreased when increasing the amount of RBCs and the obtained results were very reproducible among different experiments (Figs. 3 and 4). The binding of mycoplasma cells to RBCs was also confirmed by examining some FC samples by phase contrast and epifluorescence microscopy (Figure 4C). Since adhesion of cells from HA positive mycoplasma strains is also dependent on the RBC concentration as described previously [33], the binding reaction between both cells also follows a first order Langmuir isothermal kinetics [34] and the amount of free mycoplasmas found at any given RBC concentration can be modeled using the equation:
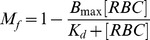
that results in an inverse Langmuir plot, where *M_f_* is the fraction of free mycoplasma cells, [*RBC*] is the number of FC events in R2 region per µL, *B*_max_ the maximum fraction of mycoplasma cells bound to RBCs and *K*_d_ is the dissociation constant of the binding reaction. Similar to other kinetic analyses performed on binding reactions involving a ligand and a receptor, the lower *K*_d_ and the higher *B*_max_ are indicative of the stronger HA activity of the tested cells.

### HA Properties of Different Mycoplasma Strains

Using this method, the HA parameters of *M. genitalium* G37 WT strain and several isogenic *M. genitalium* strains as well as two additonal mycoplasma species were determined (Figure 3A and Table 1). Obtained values from three or more biological replicates for each strain were highly reproducible. Consistent with previous works [15] G37 WT strain exhibited the lower *K*_d_ while isogenic strains mg218^−^ and mg317^−^, with an intermediate HA phenotype, showed higher *K*_d_ values, being the mg317^−^ strain *K*_d_ slightly higher than that of the WT strain. Interestingly, *B*_max_ values were very similar for WT and mg218^−^ strains and lower for mg317^−^ strain. These results suggest that the nature of the intermediate HA phenotype of strains mg218^−^ and mg317^−^ might have a different origin at the molecular level. As expected, when testing the HA negative strain mg191^−^, the number of free mycoplasmas was very high, independently of the RBC concentration and showed a non-detectable decrease even at the highest RBC concentration tested. In the absence of binding, data from strain mg191^−^ could not be modeled using the inverse Langmuir plot (Figure 3A). We also determined the HA parameters of different mycoplasma species (Figure 3B and Table 1). Cells from *M. penetrans*, a HA positive mycoplasma, exhibited a *K*_d_ value higher than *M. genitalium* cells and a *B*_max_ value not significantly lower than these cells. In contrast, cells from *M. hyopneumoniae*, a HA negative mycoplasma, showed no detectable adhesion to RBCs and binding parameters could not be determined, similarly to the non-adherent *M. genitalium* mg191^−^ strain.

**Table 1:** Hemadsorption parameters for the six strains tested.

Strain	QualitativeHA^a^	*B_max_* ± SE^b^	*K*_d_ (RBC·µL^−1^) ± SE^b^
*M. genitalium* G37	+	0.91±0.01	474.8±31.5
*M. genitalium* mg218^−^	+	0.91±0.03	1442.5±155.6
*M. genitalium* mg317^−^	+	0.68±0.04	840.8±149.4
*M. genitalium* mg191^−^	–	NA	NA
*M. penetrans* GTU-54	+	0.83±0.13	3489.4±1079.3
*M. hyopneumoniae* J	–	NA	NA

a References: *M. genitalium* G37 [40]; *M. genitalium* mg218^−^ and mg317^−^ [15]; *M. genitalium* mg191^−^ [6]; *M. penetrans* GTU-54 [41] and *M. hyopneumoniae* J [42].

b SE, standard error.

When analyzing the dot plots corresponding to different HA reactions, it was observed that mycoplasma cell aggregates with the highest SSC-H values and the strongest SYBR Green fluorescence bind to RBCs at a higher rate than single mycoplasma cells when increasing the concentration of RBCs. In addition, the presence of mycoplasma cell aggregates on RBCs was also observed in the epifluorescence images (Figure 4C). These findings were further investigated by dividing the different dot plots into four separate quartiles (Q1 to Q4, Figure 4A) and constructing inverse Langmuir plots using the fluorescence data of mycoplasma cells in each quartile. Once *K*_d_ and *B*_max_ were determined for data grouped into quartiles (Figure 4B), there was a good correlation between the HA and the size of mycoplasma cell aggregates, being the larger aggregates those that exhibited the strongest HA and providing evidence that the binding of mycoplasma to RBCs probably follows a cooperative behavior.

## Discussion

Surface-attached mycoplasmas display unique polar structures of unprecedented complexity in the bacterial world that are involved in diverse aspects of the biology of these microorganisms and play a key role as virulence factors. In addition to diverse molecular methods, attachment of mycoplasma cells has been traditionally studied by determining their HA ability to RBCs and it is a distinctive trait widely investigated when characterizing the different mycoplasma species. Despite the fact that methods to qualitatively determine the HA or hemagglutination of mycoplasmas are straightforward and inexpensive, the same is not true when investigating HA at the quantitative level [15], [21], [22], [35]. Current methods to quantify mycoplasma HA rely on centrifuging RBCs after the incubation with bacterial cells. As mycoplasma cells have a very small size, only those attached to RBCs are expected to be found in the sediment [15], [21]. However, mycoplasma cells are very prone to form large cell aggregates and some of these aggregates may sediment even at very low *g* values, introducing a bias in the results. Therefore, we choose to explore FC to quantify the HA reaction, taking advantage that this technology could resolve RBCs and mycoplasma populations in a consistent way without the need of centrifugation. Since FC separates cell populations using their light dispersion properties and fluorescence, it provides a higher resolution than centrifugation. In addition, FC quantification of SYBR Green labeled mycoplasmas is a straightforward procedure and avoids the need of introducing a previous radioactive label or determining their ATP content by expensive enzymatic methods.

As showed before, FC data are easily modeled using a kinetic approach, which allows that the HA activity of a particular mycoplasma strain could be described by standard quantitative parameters like *K*_d_ and *B*_max_. Each one of these parameters provide different information about the HA properties of tested cells. A high *K*_d_ value suggest that mycoplasma cells have a low affinity for RBCs but they are still able to adhere on their surface, especially at the highest concentrations of these cells. Likewise, a high *K*_d_ value could be expected when analyzing the HA properties of mycoplasma strains with a decreased amount of adhesins. This is in close agreement with previous reports showing that isogenic strains mg218^−^ and mg317^−^ have diminished amounts of the main adhesins P140 and P110 [15]. Alternatively, mycoplasma strains either with an improper adhesin distribution on the cell surface or bearing adhesins not properly folded are also expected to exhibit higher *K*_d_ values. On the other hand, *B*_max_ values much smaller than one are suggesting that the cell population tested is heterogeneous and a significant amount of cells are non-adherent at all, even at the highest concentration of RBCs. This may be the case of the mg317^−^ strain with a *B*_max_ value of 0.68, which indicates that 32% of cells are non-adherent and suggests that cells from this strain are heterogeneous regarding to the main adhesin content. Fortunately, this point is fully testable and work is currently in progress to assess it.

We have shown that mycoplasma aggregates bind preferentially to RBCs, suggesting a cooperative behavior when attaching to the surface of these cells. From this view, cell aggregation might be considered as a positive trait favoring the colonization of the target tissue/s. However, it is not clear whether cell aggregates are binding preferentially to the target tissues in the initial stages of an *in vivo* infection. It has been recently reported that mycoplasmas have a broad affinity for sialylated oligosaccharides [36], [37] but they show strong preferences for specific sialylated compounds, being this specificity a prominent factor to explain the marked tissue tropism that exhibit these microorganisms [38]. Bearing this in mind, the preferential attachment of cell aggregates may be simply an indication that RBC oligosaccharides promoting the binding of mycoplasmas are not as effective as those on the target tissue of a particular mycoplasma species. In this scenario, cell aggregation could be merely considered as a mechanism favoring attachment by maximizing the ratio and/or the density of adhesins on the surface of these aggregates. In addition, the preferences for particular sialylated compounds exhibited by different mycoplasma species, thus providing an explanation to the dissimilar *K*_d_ values obtained when testing the HA activity of several mycoplasma species (Figure 3B and Table 1).

In conclusion, staining mycoplasma cells with the SYBR Green vital fluorochrome combined with the FC analysis after adsorption to RBCs is an inexpensive and reliable method to accurate quantify the HA activity of mycoplasmas. This method could be easily implemented in a standardized assay to test the growing number of clinical isolates and mutant strains of different mycoplasma species, providing valuable data about the virulence of these microorganisms.

## Supporting Information

Figure S1 **RBCs and mycoplasma cells are consistently stained by SYBR Green I.** (A) RBCs analyzed in the SSC-H vs. FSC-H plot. Events were counted using the light scattering properties of RBCs. (B) Unstained RBCs in SSC-H vs FL1-H plot. Autofluorescence of unstained RBCs is extremely low and most events were found piled up in the y-axis and giving irreproducible counts. (C) SYBR Green I stained RBCs in SSC-H vs. FL1-H plot. RBCs, with a very small amount of nucleic acids, were weakly but consistently stained by SYBR Green I. When comparing values from A and C plots, 99% of events in R2 were detected after staining with SYBR Green I. (D) Unstained mycoplasma cells in SSC-H vs. FL1-H plot. Unstained mycoplasmas were slightly autofluorescent and could be enclosed in the region UR. (E) SYBR Green I stained mycoplasmas in SSC-H vs. FL1-H plot. Stained mycoplasmas were enclosed in the R1 region, which includes a very heterogeneous population of events ranging from single cells to big aggregates. A higher amount of events were detected in the stained mycoplasma sample, being the number of events in R1 consistent with the number of CFUs detected after plating the mycoplasma suspension in SP4 agar (data not shown). SYBR Green I staining also allowed to discriminate mycoplasma cells from the debris using a threshold on FL1. (F) SYBR Green I stained mycoplasma sample observed by phase contrast and epifluorescence microscopy. From 400 single cells and aggregates counted, only 3 cells (0.75%) showed no fluorescence, indicating that most of mycoplasma cells, and not only cell aggregates, are consistently stained by this procedure. Bar is 10 µm.
(DOC)

Figure S2 **Analysis of SP4 medium by Flow Cytometry.** Two samples of 50 µL of SP4 medium were diluted in 1 mL of PBSCM. The first sample was immediately stained for 20 min with SYBR Green I (panels A and B) The second sample was incubated 40 min at 37°C with end-over-end mixing and further incubated 20 min with SYBR Green I (panels C and D). All the samples were analyzed by flow cytometry using SSC-H and Fl1-H plots and regions corresponding to mycoplasmas (R1 and MR) and RBCs (R2) are marked for clarity purposes. A population of fluorescent particles is evident in all plots in a region of low complexity and low fluorescence and many of them are overlapping with R2 and R1 regions. In addition, the number of fluorescent particles increases dramatically upon incubation of SP4 medium at 37°C with mixing (panels C and D), with a significant percentage of events (13%) overlapping the mycoplasma region R1 (panel C). Since these particles are autofluorescent in the FL3 chanel, a double gating strategy based on selecting mycoplasmas in FL3-H vs FL1-H plots and including only these mycoplasma events in the new MR region was developed (panels B and D, see details in Figure 2). This double gating strategy reduces drastically the number of events from medium particles overlapping with the mycoplasma region MR (panel D).
(DOC)

Figure S3 **Flow cytometry analysis of white blood cells (WBCs).** Leukocytes from a 200 µL blood sample were purified by standard procedures using ACK buffer [39] and resuspended in 0.5 mL of PBSCM. This stock was diluted 1∶10 in PBSCM, stained with SYBR Green I and analyzed by flow cytometry (Panels A–C). In panel A, SSC and FSC laser settings were optimized for the counting of WBCs (E00 for FSC, 400 for SSC and lineal amplification). As expected, discrete populations of leukocytes were obtained in region R4 and a total of 1091 WBCs were enumerated. In panel B, the settings for SSC were also optimized for the counting of WBCs (400 for SSC) and FL1 settings were those used for mycoplasma detection (383 for FL1). A total of 1170 WBCs were detected in the region R4b and they exhibited very high FL1-H values according to their high DNA content. In panel C, the WBCs sample was analyzed using the SSC and FL1 settings optimized for mycoplasma detection (487 for SSC, 383 for FL1 and logarithmic amplification). Only 173 events of WBCs were detected with these settings in region R5, which represent 15% of the total WBCs. From these events, 4 were detected into the region of mycoplasmas MR, representing a negligible fraction of WBCs population. Cell debris from the lysis step were removed in a FSC-H versus SSC-H plot (data not shown). To compare with the previous data, a mycoplasma sample was analyzed using the same settings (Panel D). To find out the number of WBCs in the RBCs samples used to quantify the HA activity (Panels E and F), a RBCs sample was analyzed in Panel E using the same settings as in Panel B. Only 78 events of WBCs were detected in region R4b, which is a very small number when compared with the 1170 events in the WBCs preparation. When using the settings optimized for mycoplasma detection (Panel F), the number of events from WBCs in R5 was even smaller. None of these events fell into the R1 mycoplasma region. These results indicate that the small number of WBCs remaining in the RBCs preparation has no effect when quantifying the mycoplasma cell biomass after the hemadsorption reaction.
(DOC)

Figure S4 **Flow cytometry (FC) analyses of Mycoplasma non-viable cells.** Three *Mycoplasma genitalium* cell samples were prepared at a 3·10^6^ FL1 fluorescence units mL^−1^ (the usual working dilution for FC analyses, see main text). These samples were incubated 40 min at 37°C with end-over-end mixing and stained 20 min with SYBR Green I. To detect the presence of non-viable cells, some of the samples were also stained with 2 µg mL^−1^ propidium iodide (PI) for 5 min before being analyzed by FC using SSC-H vs. FL1-H plots (panels A, C and E) and FL3-H vs. FL1-H plots (panels B, D and F). Since PI staining increases dramatically the FL3-H fluorescence of dead mycoplasma cells, the double gating strategy to reduce the number of events from medium particles overlapping with the mycoplasma region R1 could not be used. Alternatively, the mycoplasma population region R1 was delimited in SSC-H vs. FL1-H plots and this region was then gated in FL3-H vs. FL1-H plots. Panels A-B: control sample containing mycoplasma cells non-stained with PI. Panels C-D: positive control containing PI-stained cells permeabilized with 0.015% Triton X-100 to demonstrate that non-viable cells are strongly stained with PI and exhibit a concomitant increase in FL3-H fluorescence. PI-stained cells could be enclosed in a region DR. Panels E–F: unpermeabilized mycoplasma cells stained with PI. FC analyses showed a slight increase in FL3-H fluorescence and only a small fraction of the events (4.3%) fell into the DR region, suggesting that most of the mycoplasma cells remain viable in the conditions used to perform the HA assay.
(DOC)

Figure S5 **HA assay using a fixed amount of RBCs and increasing amounts of mycoplasma cells.** (+) column contains the dot plots of the mixtures of RBCs and increasing amounts of mycoplasmas. In (−) column are the dot plots of the same mycoplasma samples in the absence of RBCs, respectively. (A–D) When increasing the mycoplasma concentration from A to D in the hemadsorption reaction, the fluorescence in the RBC R2 region shifts to higher FL1-H fluorescence values as a consequence of the attachment of mycoplasmas. Despite the fact that the data could be modeled to a Langmuir plot, medium debris invade the R2 region as seen in the (−) column and, as a result, R2 FL1-H total fluorescence measures are not reliable. Furthermore, when using the highest amounts of mycoplasma cells (C–D) cell aggregates overlap to the R2 region and RBCs containing attached mycoplasma invades the R1 region, making difficult to obtain reproducible measures of RBCs in R2 region.
(DOC)

Protocol S1 **Detailed working protocol of the whole HA assay procedure.**
(DOC)
