# Supplementary material for: Quantitative Assessment of Mycoplasma Hemadsorption Activity by Flow Cytometry
Source: PLoS One. 2014 Jan 30;9(1):e87500. doi: 10.1371/journal.pone.0087500 (PMC3907496; doi:10.1371/journal.pone.0087500)
Supplement: Figure S1 — RBCs and mycoplasma cells are consistently stained by SYBR Green I. (A) RBCs analyzed in the SSC-H vs. FSC-H plot. Events were counted using the light scattering properties of RBCs. (B) Unstained RBCs in SSC-H vs FL1-H plot. Autofluorescence of unstained RBCs is extremely low and most events were found piled up in the y-axis and giving irreproducible counts. (C) SYBR Green I stained RBCs in SSC-H vs. FL1-H plot. RBCs, with a very small amount of nucleic acids, were weakly but consistently stained by SYBR Green I. When comparing values from A and C plots, 99% of events in R2 were detected after staining with SYBR Green I. (D) Unstained mycoplasma cells in SSC-H vs. FL1-H plot. Unstained mycoplasmas were slightly autofluorescent and could be enclosed in the region UR. (E) SYBR Green I stained mycoplasmas in SSC-H vs. FL1-H plot. Stained mycoplasmas were enclosed in the R1 region, which includes a very heterogeneous population of events ranging from single cells to big aggregates. A higher amount of events were detected in the stained mycoplasma sample, being the number of events in R1 consistent with the number of CFUs detected after plating the mycoplasma suspension in SP4 agar (data not shown). SYBR Green I staining also allowed to discriminate mycoplasma cells from the debris using a threshold on FL1. (F) SYBR Green I stained mycoplasma sample observed by phase contrast and epifluorescence microscopy. From 400 single cells and aggregates counted, only 3 cells (0.75%) showed no fluorescence, indicating that most of mycoplasma cells, and not only cell aggregates, are consistently stained by this procedure. Bar is 10 µm. (DOC) [file pone.0087500.s001.doc]

**Figure S1**

**
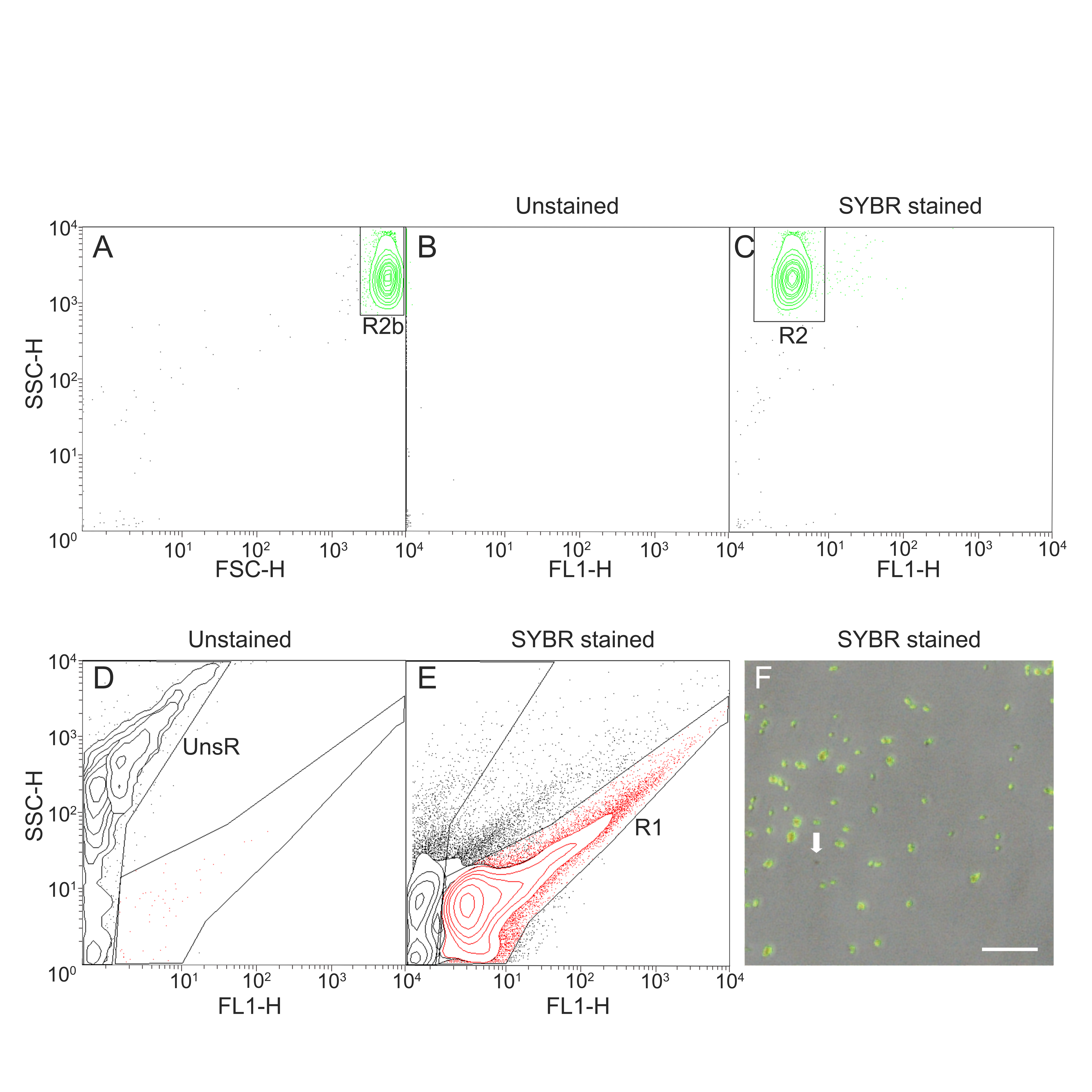
**


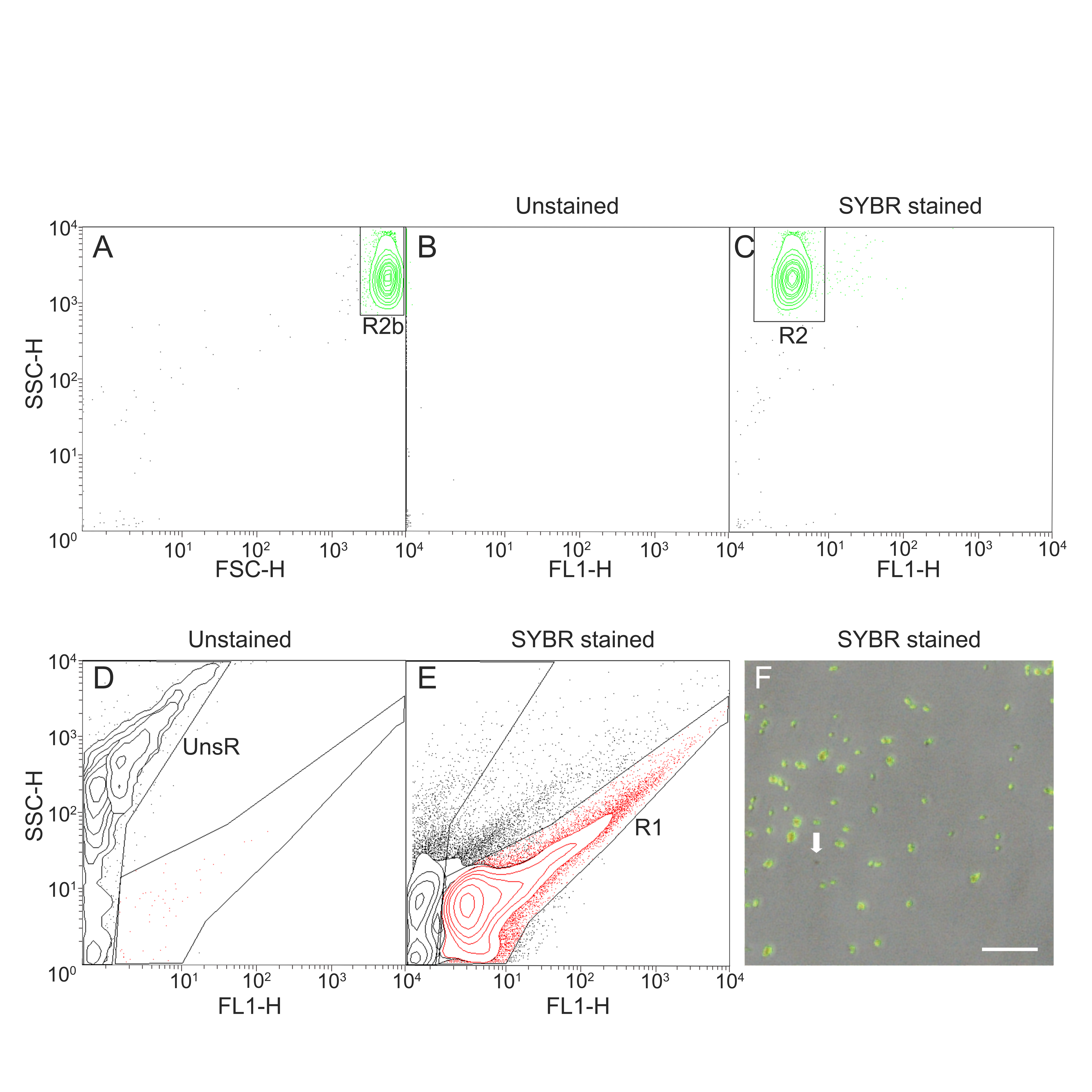


4573 events

4540 events

| ***M. genitalium* sample** | **events in UR** | **events in R1** |
| --- | --- | --- |
| Unstained | 1528 (96.6%) | 54 (3.4%) |
| SYBR Green I stained | 12253 (23%) | 40958 (77%) |
