# Supplementary figures and images for: Quantitative Assessment of Mycoplasma Hemadsorption Activity by Flow Cytometry
Source: PLoS One. 2014 Jan 30;9(1):e87500. doi: 10.1371/journal.pone.0087500 (PMC3907496; doi:10.1371/journal.pone.0087500)

**Figure S2**


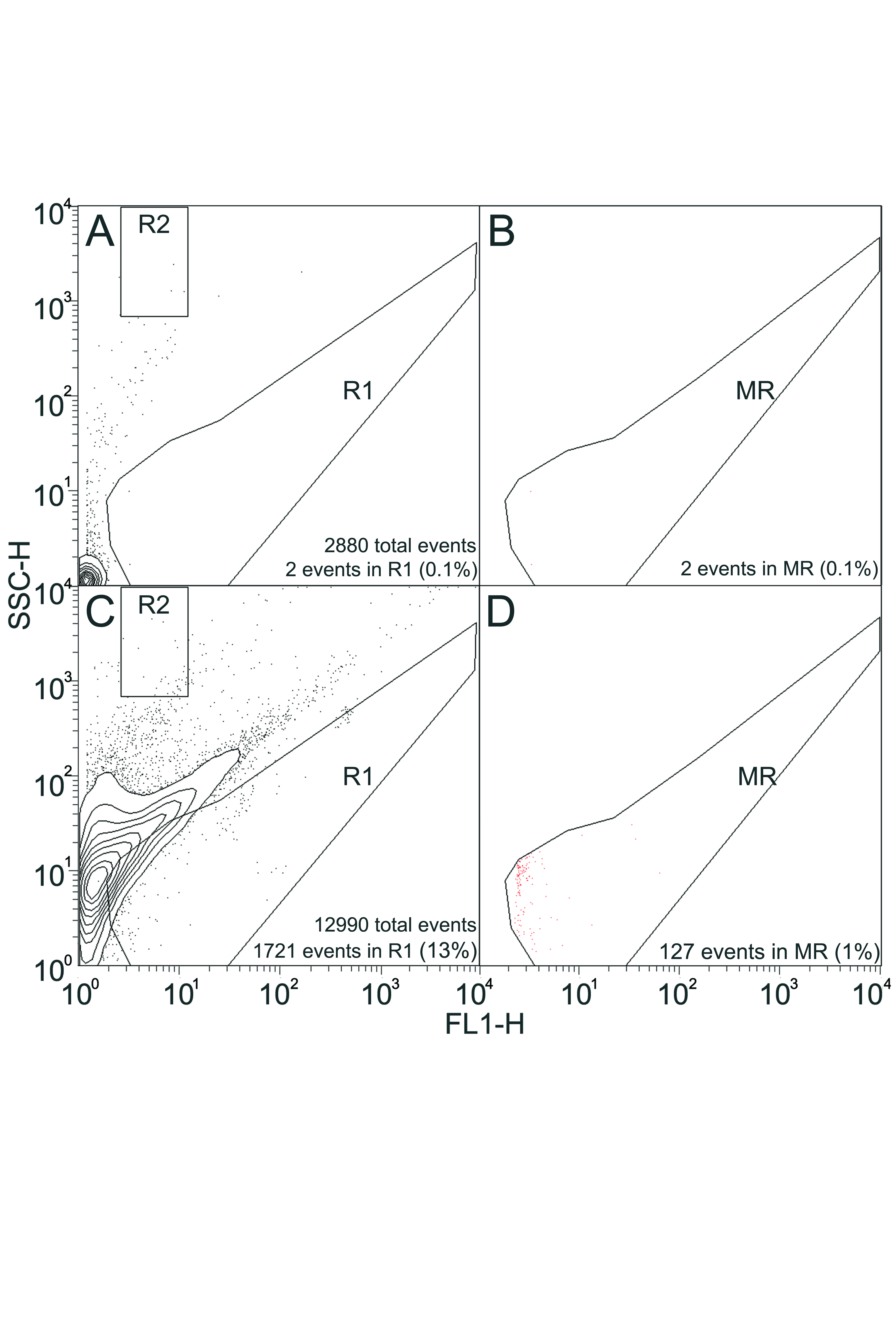

Supplement: Figure S2 — Analysis of SP4 medium by Flow Cytometry. Two samples of 50 µL of SP4 medium were diluted in 1 mL of PBSCM. The first sample was immediately stained for 20 min with SYBR Green I (panels A and B) The second sample was incubated 40 min at 37°C with end-over-end mixing and further incubated 20 min with SYBR Green I (panels C and D). All the samples were analyzed by flow cytometry using SSC-H and Fl1-H plots and regions corresponding to mycoplasmas (R1 and MR) and RBCs (R2) are marked for clarity purposes. A population of fluorescent particles is evident in all plots in a region of low complexity and low fluorescence and many of them are overlapping with R2 and R1 regions. In addition, the number of fluorescent particles increases dramatically upon incubation of SP4 medium at 37°C with mixing (panels C and D), with a significant percentage of events (13%) overlapping the mycoplasma region R1 (panel C). Since these particles are autofluorescent in the FL3 chanel, a double gating strategy based on selecting mycoplasmas in FL3-H vs FL1-H plots and including only these mycoplasma events in the new MR region was developed (panels B and D, see details in Figure 2). This double gating strategy reduces drastically the number of events from medium particles overlapping with the mycoplasma region MR (panel D). (DOC) [file pone.0087500.s002.doc]

**Figure S4**


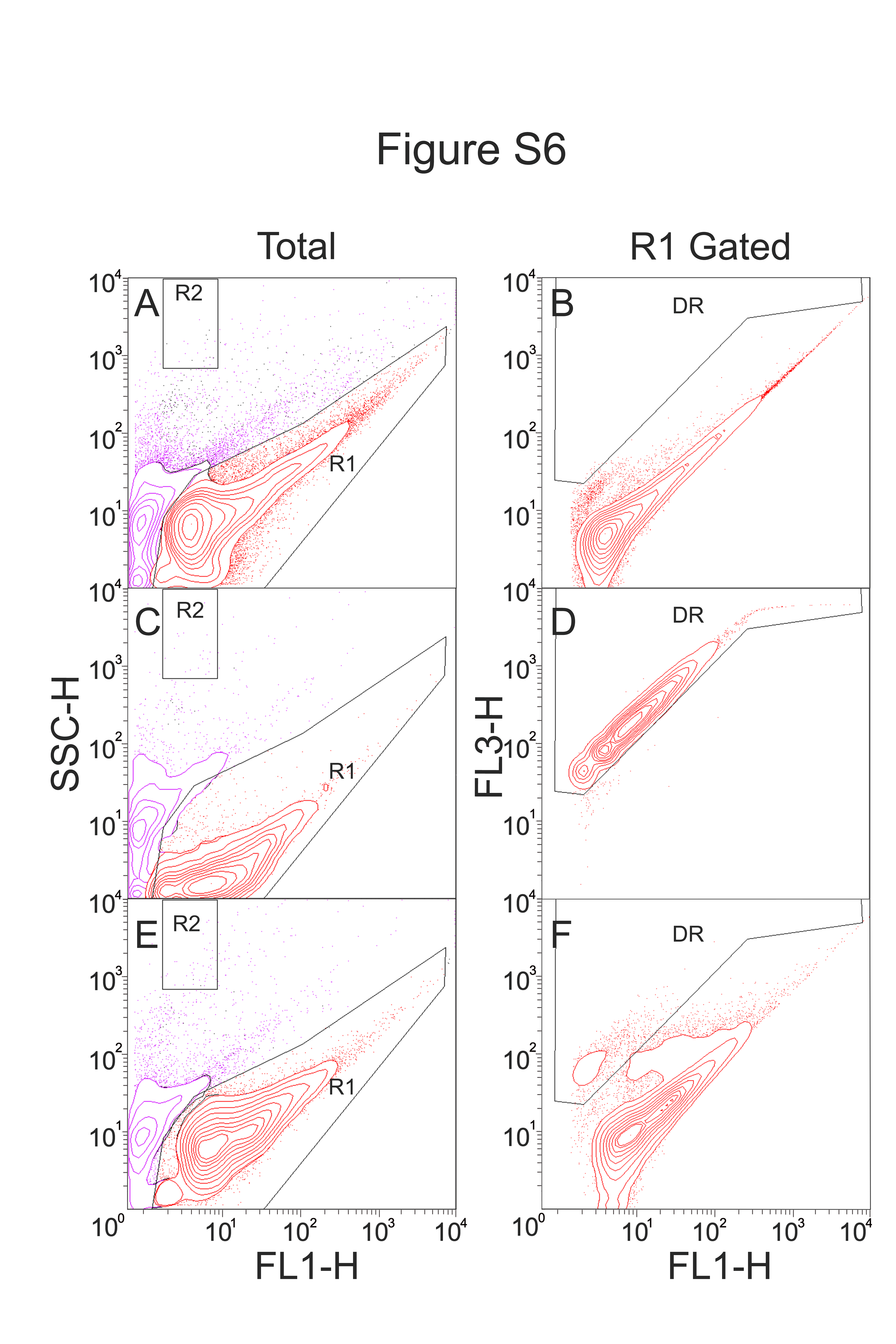


| Treatment | Cells in R1 (% of gate) | Cells in DR (% gate) |
| --- | --- | --- |
| No treatment | 20709 (73.8%) | 10 (0.05%) |
| PI + Triton X-100 | 4716 (68.5%) | 4648 (98.6%) |
| PI | 18485 (80.7%) | 789 (4.3%) |

Supplement: Figure S4 — Flow cytometry (FC) analyses of Mycoplasma non-viable cells. Three Mycoplasma genitalium cell samples were prepared at a 3·106 FL1 fluorescence units mL−1 (the usual working dilution for FC analyses, see main text). These samples were incubated 40 min at 37°C with end-over-end mixing and stained 20 min with SYBR Green I. To detect the presence of non-viable cells, some of the samples were also stained with 2 µg mL−1 propidium iodide (PI) for 5 min before being analyzed by FC using SSC-H vs. FL1-H plots (panels A, C and E) and FL3-H vs. FL1-H plots (panels B, D and F). Since PI staining increases dramatically the FL3-H fluorescence of dead mycoplasma cells, the double gating strategy to reduce the number of events from medium particles overlapping with the mycoplasma region R1 could not be used. Alternatively, the mycoplasma population region R1 was delimited in SSC-H vs. FL1-H plots and this region was then gated in FL3-H vs. FL1-H plots. Panels A-B: control sample containing mycoplasma cells non-stained with PI. Panels C-D: positive control containing PI-stained cells permeabilized with 0.015% Triton X-100 to demonstrate that non-viable cells are strongly stained with PI and exhibit a concomitant increase in FL3-H fluorescence. PI-stained cells could be enclosed in a region DR. Panels E–F: unpermeabilized mycoplasma cells stained with PI. FC analyses showed a slight increase in FL3-H fluorescence and only a small fraction of the events (4.3%) fell into the DR region, suggesting that most of the mycoplasma cells remain viable in the conditions used to perform the HA assay. (DOC) [file pone.0087500.s004.doc]

**Figure S5**

**
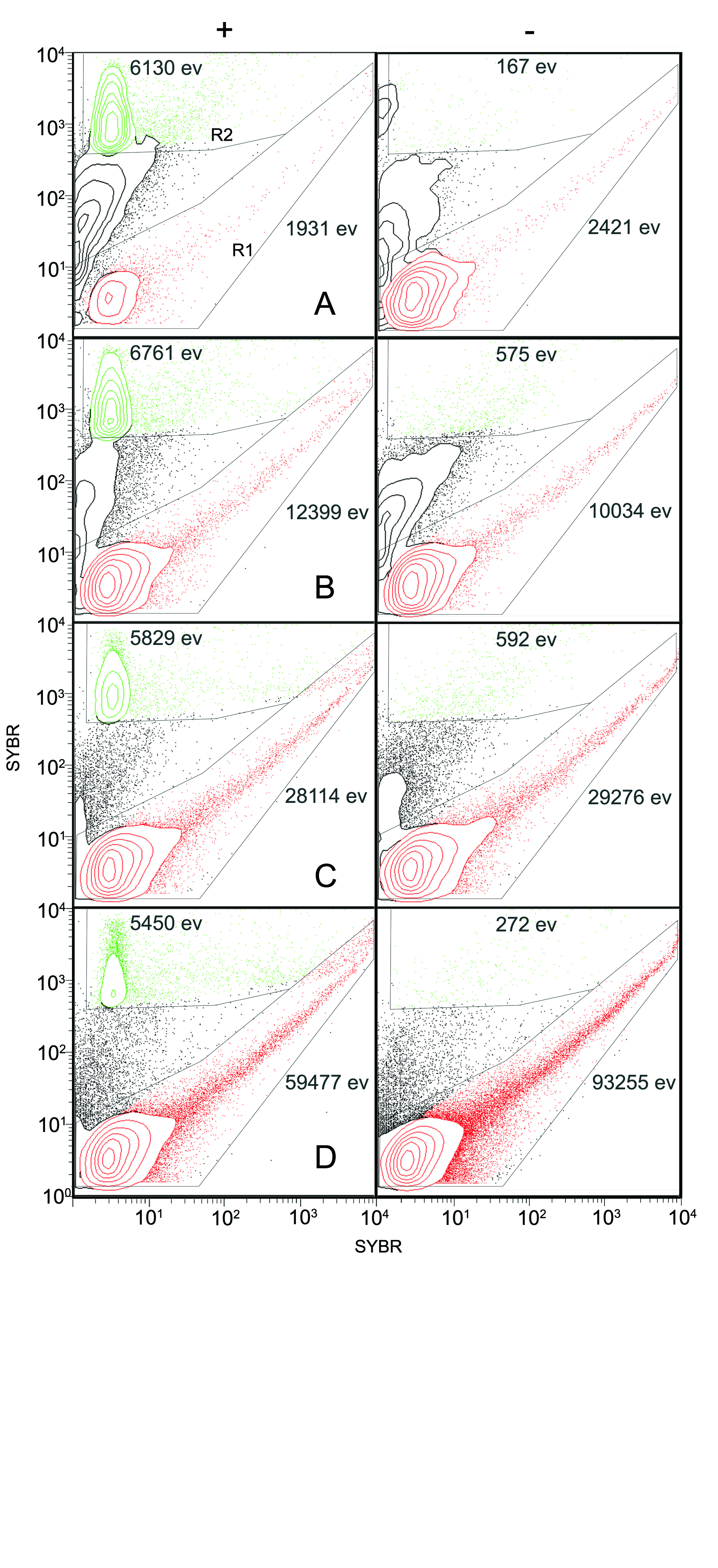
**

SSC-H

FL1-H

Supplement: Figure S5 — HA assay using a fixed amount of RBCs and increasing amounts of mycoplasma cells. (+) column contains the dot plots of the mixtures of RBCs and increasing amounts of mycoplasmas. In (−) column are the dot plots of the same mycoplasma samples in the absence of RBCs, respectively. (A–D) When increasing the mycoplasma concentration from A to D in the hemadsorption reaction, the fluorescence in the RBC R2 region shifts to higher FL1-H fluorescence values as a consequence of the attachment of mycoplasmas. Despite the fact that the data could be modeled to a Langmuir plot, medium debris invade the R2 region as seen in the (−) column and, as a result, R2 FL1-H total fluorescence measures are not reliable. Furthermore, when using the highest amounts of mycoplasma cells (C–D) cell aggregates overlap to the R2 region and RBCs containing attached mycoplasma invades the R1 region, making difficult to obtain reproducible measures of RBCs in R2 region. (DOC) [file pone.0087500.s005.doc]
