# Supplementary material for: Quantitative Assessment of Mycoplasma Hemadsorption Activity by Flow Cytometry
Source: PLoS One. 2014 Jan 30;9(1):e87500. doi: 10.1371/journal.pone.0087500 (PMC3907496; doi:10.1371/journal.pone.0087500)
Supplement: Figure S3 — Flow cytometry analysis of white blood cells (WBCs). Leukocytes from a 200 µL blood sample were purified by standard procedures using ACK buffer [39] and resuspended in 0.5 mL of PBSCM. This stock was diluted 1∶10 in PBSCM, stained with SYBR Green I and analyzed by flow cytometry (Panels A–C). In panel A, SSC and FSC laser settings were optimized for the counting of WBCs (E00 for FSC, 400 for SSC and lineal amplification). As expected, discrete populations of leukocytes were obtained in region R4 and a total of 1091 WBCs were enumerated. In panel B, the settings for SSC were also optimized for the counting of WBCs (400 for SSC) and FL1 settings were those used for mycoplasma detection (383 for FL1). A total of 1170 WBCs were detected in the region R4b and they exhibited very high FL1-H values according to their high DNA content. In panel C, the WBCs sample was analyzed using the SSC and FL1 settings optimized for mycoplasma detection (487 for SSC, 383 for FL1 and logarithmic amplification). Only 173 events of WBCs were detected with these settings in region R5, which represent 15% of the total WBCs. From these events, 4 were detected into the region of mycoplasmas MR, representing a negligible fraction of WBCs population. Cell debris from the lysis step were removed in a FSC-H versus SSC-H plot (data not shown). To compare with the previous data, a mycoplasma sample was analyzed using the same settings (Panel D). To find out the number of WBCs in the RBCs samples used to quantify the HA activity (Panels E and F), a RBCs sample was analyzed in Panel E using the same settings as in Panel B. Only 78 events of WBCs were detected in region R4b, which is a very small number when compared with the 1170 events in the WBCs preparation. When using the settings optimized for mycoplasma detection (Panel F), the number of events from WBCs in R5 was even smaller. None of these events fell into the R1 mycoplasma region. These results indicate that the small number of WBCs remainin [file pone.0087500.s003.doc]

**Figure S3**


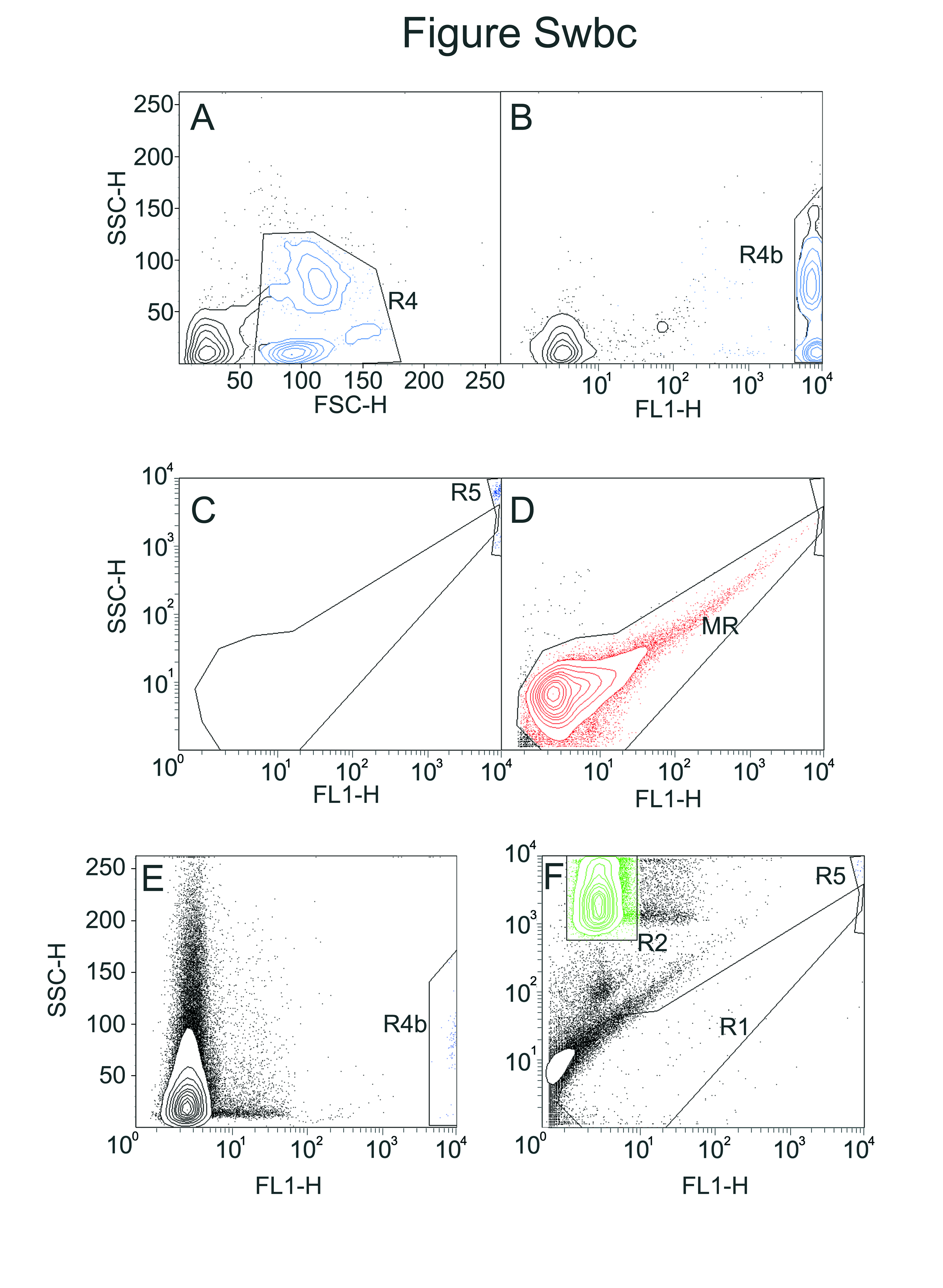


| **Region** | **Cell type** | **Number of events**  **(% of cell type)** |
| --- | --- | --- |
| Flow cytometry analysis of white blood cells (WBCs). Panels A, B and C. | | |
| R4 | WBCs | 1091 (93%) |
| R4b | WBCs | 1170 (100%) |
| R5 | WBCs | 173 (15%) |
| R5 and MR | WBCs | 4 (0.3%) |
| Flow cytometry analysis *M. genitalium*. Panel D. | |  |
| MR | *M. genitalium* G37 | 41991 (100%) |
| R5 and MR | *M. genitalium* G37 | 1 (0.002%) |
| Flow cytometry analysis of RBCs with added SP4 medium. Panels E, F. | | |
| R4b | WBCs | 78 (100%) |
| R5 | WBCs | 16 (20%) |
| R5 and R1 | WBCs | 0 (0%) |
| R2 | RBCs | 141845 (100%) |
| R1 | SP4 Debris | 350 |
