# Supplementary material for: Quantitative Assessment of Mycoplasma Hemadsorption Activity by Flow Cytometry
Source: PLoS One. 2014 Jan 30;9(1):e87500. doi: 10.1371/journal.pone.0087500 (PMC3907496; doi:10.1371/journal.pone.0087500)
Supplement: Protocol S1 — Detailed working protocol of the whole HA assay procedure. (DOC) [file pone.0087500.s006.doc]

**Protocol S1**

**Working Protocol: Flow Cytometry analysis of the Hemadsorption Activity of Mycoplasma Strains**

**Part 1: Preparation of Biological Stocks**

1. Preparation of Mycoplasma Stock
   - Grow the desired strain of mycoplasma in 20 mL of culture medium (SP4, Friis, Hayflick...). Adherent strains are usually grown in tissue culture flasks. Start one culture for each biological repeat.
   - Recover the cells at the end of the exponential phase of grow. This can be assessed by the color change of the culture medium. If the mycoplasma strain is adherent, cells should be scrapped off the flasks. Wash mycoplasma cells by centrifugation at 20 000 *g* 20 min.
   - Resuspend the pellets in 0.5 mL of the same culture media. If using Hayflick media, use a medium stock without DNA. DNA in the culture medium will stain also with SYBR Green I, interfering with the detection of mycoplasmas by flow cytometry (FC).
   - Pass the samples 10 times through a 25 G syringe. This step is essential to minimize the number of cell aggregates.
   - Store the mycoplasma samples on ice.
2. Preparation of the Stock of Red Blood Cells (RBCs)
   - For convenience, we are using human RBCs but RBCs from animal origin (sheep, pig, etc) can be used. Since the amount of RBCs used in the HA assay is very small, 5-6 drops (about 0.250 mL) of peripheral blood will suffice. To obtain RBCs from human origin we are using sterile Ames Minilet^TM^ Lancets (Bayer). Whatever will be origin of RBCs, remember that you need the approval of the procedure used by the corresponding Committee of Human and Animal Experimentation of your institution

- Wash 0.250 mL of the extracted blood 3 times with 10 mL of PBSCM (PBS with calcium chloride and magnesium chloride, Sigma-aldrich corp.). Centrifugate RBCs 2 min at 300 g to remove the supernatant.
- Dilute the washed RBCs in 25 mL of PBSCM and store on ice.

**Part 2: Titration of the Biological Stocks**

1. Titration of the Stocks of Mycoplasma Cells

- Dilute 1:200 an aliquot of the mycoplasma stock in culture medium.
- In a 1.5 mL eppendorf tube, dilute 20 µL of the 1:200 mycoplasma dilution in 500 µL of PBSCM. Simultaneously, prepare another sample with 480 µL of PBSCM and 20 µL of the culture medium as negative control.
- Add 5 µL of SYBR Green I diluted 1:100 from the commercial stock (Molecular Probes) in PBSCM in both samples and mix by inversion.
- Incubate the samples 20 min at dark and room temperature
- Read the sample in a FC device. We are using a FACSCalibur (Becton Dickinson, USA) equipped with an air-cooled 488 nm argon laser and a 633 nm red diode laser. Readings were performed using side-angle-scatter (SSC-H), green fluorescence (FL1-H detector, 530/30 filter) and red autofluorescence (FL3-H detector, 670LP filter). The primary threshold was set at 11 units of SSC-H and the secondary threshold at 27 units of FL1-H. The flow rate used was 12 µL s^-1^. A dual plot of SSC-H over FL1-H fluorescence allows limiting a mycoplasma cells characteristic region (R1, Fig 1A in the paper).

Note: Using a threshold at SSC-H and FL1-H allows eliminating events from components of the culture medium.

- The total fluorescence obtained when multiplying the number events by the FL1-H mean of the R1 region is a measure the mycoplasma cell mass once subtracted the total fluorescence of this region in the negative control sample.

1. Titration of the RBCs stock

- Dilute 1 mL of the RBCs stock in 15 mL of PBSCM.
- In a 1.5 mL eppendorf tube, dilute 50 µL of the previous dilution in 500 µL of PBSCM. Prepare also another sample of 500 µL of PBSCM without RBCs that will be used as negative control.
- Add 5 µL of SYBR Green I diluted 1:100 from the commercial stock in PBSCM in both samples and mix by inversion.
- Incubate the samples in the dark for 20 min at room temperature.
- Read the samples in a FC device using the same settings to titter the mycoplasma samples. A dual plot of SSC-H over FL1-H allows limiting a RBCs characteristic region (R2, figure 1A).
- Events in R2 region indicates the RBCs concentration when subtracted the number of events in the corresponding region of the negative control.


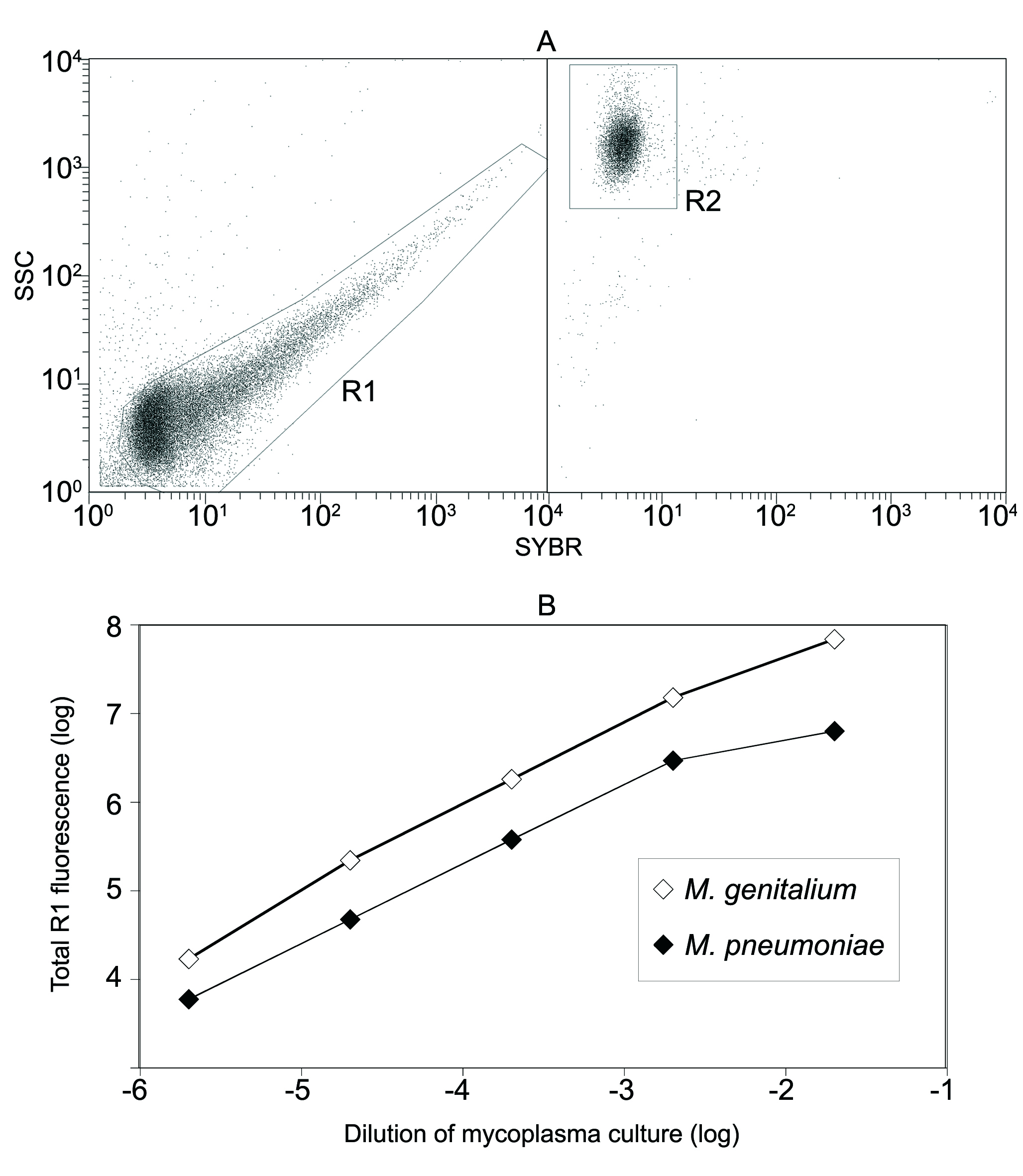


**FL1-H**

**Part 3: Hemadsorption reaction**

- Dilute an aliquot of the RBCs stock to 2·10^7^ events·mL^-1^ in PBSCM.
- Dilute an aliquot of the mycoplasma stock to 1·10^8^ units of total FL1-H fluorescence·per mL.

Note: the units of fluorescence may vary in different FC devices and lasers. However, the amount of mycoplasmas used in the experiment is not critical if this number is into the linear range (Fig 1B in the paper).

- Prepare a dilution bank in 1 mL of final volume with PBSCM, 30 μL of the mycoplasma aliquot (about 3·10^7^ units of total FL1-H fluorescence mL^-1^), 20 μL of culture medium and the desired amount of RBC from 0 to 10^7^ RBC·mL^-1^. For example:

| Tube | PBSCM (µL) | RBC (µL) | Medium (µL) | Mycoplasma (µL) |
| --- | --- | --- | --- | --- |
| 1 | 947 | 3 | 20 | 30 |
| 2 | 940 | 10 | 20 | 30 |
| 3 | 920 | 30 | 20 | 30 |
| 4 | 890 | 60 | 20 | 30 |
| 5 | 850 | 100 | 20 | 30 |
| 6 | 750 | 200 | 20 | 30 |
| 7 | 650 | 300 | 20 | 30 |
| 0 | 950 | 0 | 20 | 30 |

Note: the 0 sample is used to calculate the actual number of mycoplasma cells in the experiment.

- Prepare also the same dilution bank without mycoplasmas, adding the corresponding volume of the culture medium. This will be used as negative control as in part 2.
- Incubate the samples 40 min at 37ºC in a tube rotator device providing end-over-end mixing.

Note: it is recommended to use rounded 2 mL centrifugation tubes to avoid RBCs sedimentation.

- Add 10 µL of 1:100 diluted SYBR green-1 in PBSCM and incubate in the dark for 20 min at room temperature.
- Read the samples in the FC device. A dual plot of FL3-H over FL1-H allows separating mycoplasma cells in a R3 region (Fig 2B in the paper).
- Perform a second plot of SSC-H over FL1-H only with events from R3 to resolve the free mycoplasma cells (MR, Fig 2C in the paper) from the RBCs containing attached mycoplasma cells.

**Part 4: Plotting the results and obtaining HA parameters**

- For each tube of the dilution bank, convert the total fluorescence in MR into fraction of free mycoplasma cells dividing by the MR total fluorescence of the 0 tube.

Note: subtract the total fluorescence in MR of each tube with the total fluorescence in MR in the corresponding negative control

- Convert events in the region of RBCs (R1) into events·µL^-1^.

Note: It is easier to count RBCs events in the samples of the negative control. In the absence of mycoplasma cells, RBCs are enclosed in R1 region and do not aggregate.

- Represent the fraction of free mycoplasma over the concentration of RBCs. Fit to an inverted Langmuir isotherm by iteration, determining in this way the *K_d_* and *B_max_* constants. We perform the fitting using the next equation, which can be easily implemented in the Kaleidagraph software:

$$M_{f}=1-\frac{B_{\max}\left[RBC\right]}{K_{d}+\left[RBC\right]}$$
